# Genome-wide expression links the electron transfer pathway of *Shewanella oneidensis *to chemotaxis

**DOI:** 10.1186/1471-2164-11-319

**Published:** 2010-05-21

**Authors:** Shang-Kai Tai, GuanI Wu, Shinsheng Yuan, Ker-Chau Li

**Affiliations:** 1 Institute of Statistical Science, Academia Sinica, Taipei 115, Taiwan; 2 Department of Statistics, University of California, Los Angeles, CA 90095-1554, USA

## Abstract

**Background:**

By coupling the oxidation of organic substrates to a broad range of terminal electron acceptors (such as nitrate, metals and radionuclides), *Shewanella oneidensis *MR-1 has the ability to produce current in microbial fuel cells (MFCs). *omcA*, *mtrA*, *omcB *(also known as *mtrC*), *mtrB*, and *gspF *are some known genes of *S. oneidensis *MR-1 that participate in the process of electron transfer. How does the cell coordinate the expression of these genes? To shed light on this problem, we obtain the gene expression datasets of MR-1 that are recently public-accessible in Gene Expression Omnibus. We utilize the novel statistical method, liquid association (LA), to investigate the complex pattern of gene regulation.

**Results:**

Through a web of information obtained by our data analysis, a network of transcriptional regulatory relationship between chemotaxis and electron transfer pathways is revealed, highlighting the important roles of the chemotaxis gene *cheA-1*, the magnesium transporter gene *mgtE-1*, and a triheme *c*-type cytochrome gene SO4572.

**Conclusion:**

We found previously unknown relationship between chemotaxis and electron transfer using LA system. The study has the potential of helping researchers to overcome the intrinsic metabolic limitation of the microorganisms for improving power density output of an MFC.

## Background

*Shewanella oneidensis *MR-1 (= ATCC 700550 = CIP 106686 = BCRC 17276), previously designated *Alteromonas putrefaciens *MR-1 or *Shewanella putrefaciens *strain MR-1, is a facultative anaerobic gram-negative bacterium with a single unsheathed polar flagellum [1-4]. The strain MR-1, isolated from Oneida Lake in New York, shows bioremediation potential and metabolically versatile properties. Under aerobic conditions, *S. oneidensis *utilizes oxygen as the final electron acceptor; nevertheless, *S. oneidensis *undertakes respiration by reducing alternative terminal electron acceptors such as nitrite, sulfite, fumarate, metals [Mn(III/IV), Fe(III), and Cr(VI)], and radionuclides [U(VI) and Pu(IV)] under anaerobic environment [5-10]. The remarkable anaerobic respiratory plasticity (ARP) involves many genes. In this study, we only considered a subset, *cymA*, *mtrA*, *mtrB*, *omcB *(also known as *mtrC*), *omcA*, *gspF*, and *gspD *genes [11,12].

The functions of these ARP genes have been characterized. The gene *cymA *(locus tag SO4591) encodes a cytoplasmic membrane-bound, tetraheme cytochrome *c *that serves as an entry point for electron flow from the cytoplasm to decaheme cytochrome *c*, encoded by *mtrA *(SO1777) [11-13]. The electrons are relayed through the periplasm to the outer membrane (OM) protein encoded by the gene *mtrB *(SO1776) [11-13], which also plays a role required for the proper localization and insertion of cytochromes OmcB (SO1778) and OmcA (SO1779) into the OM [11,13,14]. OmcB interacts directly with OmcA to form a stable complex as part of the electron transport pathway [11,13,15]. OmcA is a cell surface-exposed lipoprotein, that has been shown to be involved in the process of electron transfer to electrodes in microbial fuel cells (MFCs) [11,13,16,17]. On the cell surface, exposure of the OmcA allows it to directly contact with extracellular electron acceptors [16]. Both genes *gspF *(SO0168) and *gspD *(SO0166) encode individual components of the type II secretion system (T2S). Pseudopilus apparatus of T2S, whose formation can be regulated by GspF, delivers OmcB and OmcA from periplasm across GspD into the surroundings where the OmcB-OmcA complex is constructed [12,18].

We are interested in studying how the cell coordinate the expression of these seven ARP genes. There are a total of 4,931 predicted protein-encoding open reading frames (CDSs) in *S. oneidensis *MR-1, comprising a circular chromosome and an iteron-type plasmid with 4,758 and 173 CDSs respectively [7]. Genome-wide gene expression profiling has been a powerful method in elucidating the gene regulation patterns in cells [19,20]. For example, the well-known yeast gene expression dataset [21], originally collected for finding cell cycle-regulated genes, has been used by some authors to study biological mechanisms beyond the cell-cycle events [22-24]. Inspired by such successes, we searched NCBI Gene Expression Omnibus [25] for experiments performed on the strain MR-1 and found three such datasets, series GSE3876, GSE4489, and GSE7973. They were generated by the spotted cDNA microarray method. We combined these three gene expression datasets to form a full dataset (denoted by gpl3253_cia) with 88 conditions for investigation. Our aim is to study how the expression of the aforementioned genes of electron transfer are coregulated and how they may interact with other genes. We employed the new bioinformatic tool, liquid association (LA), to conduct the data analysis [22,26-28].

LA can be viewed as an extension of the traditional correlation measure which is commonly employed in gene expression studies for identifying gene clusters. Genes with similar expression profiles, as reflected by significant correlation coefficient, tend to form common structure components, to be regulated by common transcription elements, and to participate in the same biological pathways. However, many functionally associated genes are uncorrelated in expression [29]. LA is a method for identifying higher order association between variables in complex systems. It is particularly useful when the correlation between two variables X, Y is weakened due to the mediation by a third variable Z. LA depicts how the pattern of correlation between X and Y, including its sign and strength, is mediated by Z.

We uploaded gpl3253_cia to the LA online computing system. We used each pair of the ARP genes as the lead, X and Y, to generate a short list of genes Z with the highest LA scores. Through the genes which mediate the correlation patterns of ARP genes, we hope to unravel some biological pathways important to electron transfer process. After examining the LA output, one gene *cheA-1 *stood out from a pool of near 5000 genes in the genome as the best LA score gene. Examination of the genome of MR-1 showed that this bacterium has two uninterrupted **che**motaxis (*che*) genes, designated *cheA-1 *(SO2121) and *cheA-3 *(SO3207) [7,30,31]. CheA (a histidine protein kinase), together with CheW and CheZ can control the level of phosphorylation of CheY, which regulates flagellar motion [32]. On the other hand, at least five studies had shown that MR-1 responds chemotactically to a wide range of anaerobic electron acceptors [31,33-36]. In particular, Baraquet *et al*. showed that the anaerobic respiratory systems are necessary for chemotaxis towards anaerobic electron acceptors [36]. In addition, *cheA-3 *gene was demonstrated to be essential for the chemotactic behavior in MR-1 [31]. Putting together, our results suggest a mediating role for *cheA-1 *in the chemotactic responses to anaerobic electron acceptors. Encouraged by this finding, we further conducted a series of LA analysis and reported additional results for *cheA *and other genes.

## Results

**Figure 1:**
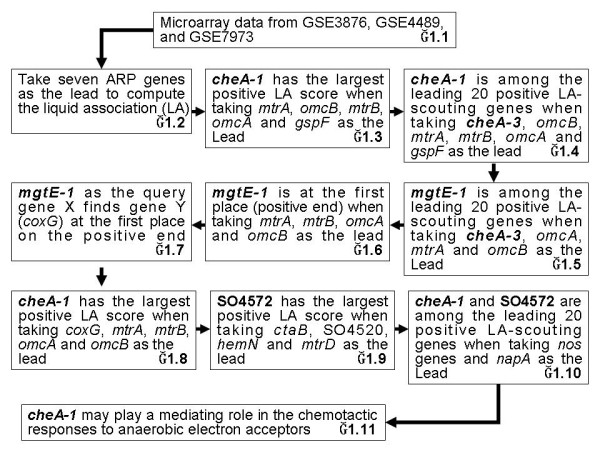
**A schematic chart of a series of LA analyses conducted in this study**. The findings highlight three most significant LA-scouting genes *cheA-1*, *mgtE-1 *and SO4572, that affect many pairs of the anaerobic respiration genes.

**Figure 2:**
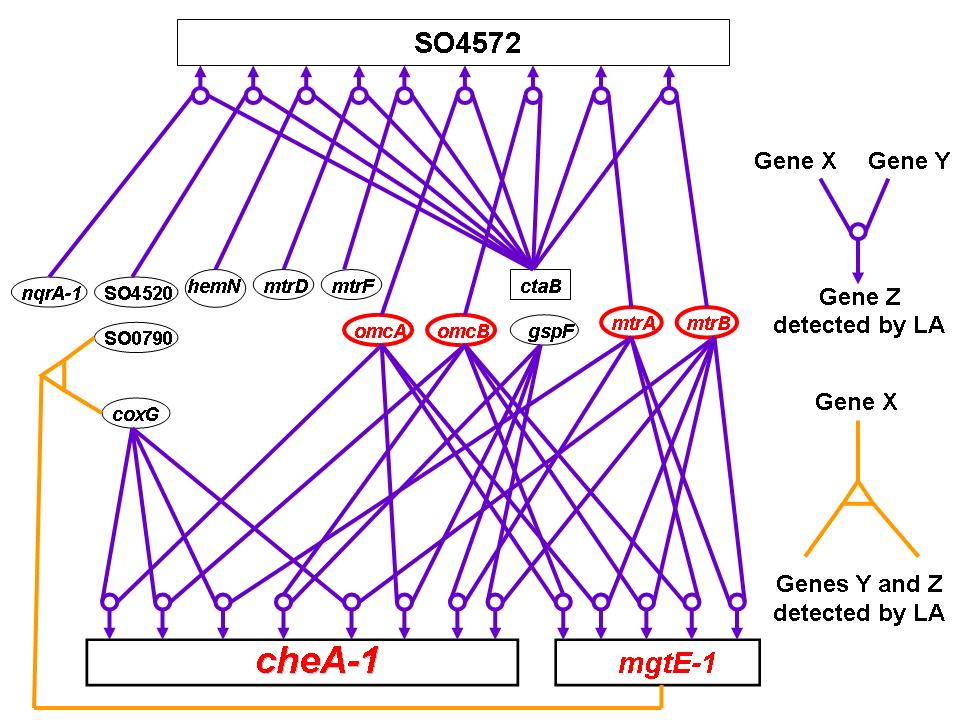
**Liquid association (LA) network analysis**. The LA-scouting genes are occupied at the first place on the positive end of best LA score gene list. The purple arrows point to the mediator genes under the LA score system with X and Y genes as the input. When only one gene X = *mgtE-1 *is given as the input gene, we can find that the gene pair Y = *coxG*, Z = SO0790 appears at the first place on the positive score end (the orange line).

We have conducted a series of LA analysis as depicted in Figure 1. Our findings can be summarized by Figure 2, which shows three most significant LA-scouting genes *cheA-1*, *mgtE-1 *and SO4572, that affect many pairs of the anaerobic respiration genes. The chemotaxis gene *cheA-1 *is already discussed earlier. The second gene *mgtE-1 *found in our LA analysis encodes a magnesium transporter, suggesting a possible connection between electron transfer and the magnesium transport system in *S. oneidensis*. This is consistent with a recent study on cobalt reduction wherein the authors found not only the critical involvement of the Mtr respiratory proteins (including MtrA, MtrB, OmcB) but also pointed out that the process could be influenced by magnesium concentrations [37]. Furthermore, the ability of electricity production and Fe(III) reduction in *S. oneidensis *is similar to that in the bacterium *Aeromonas hydrophila *[38], of which some *mgtE *mutants showed significantly reduced swarming in semisolid swarming agar [39]. Chemotaxis is essential for swarming motility in bacteria [40]. Thus our finding brings out a likely coordinative gene regulation between the chemotaxis pathway, the electron transfer and the magnesium transport system. SO4572 encodes a triheme *c*-type cytochrome. A deletion mutant of SO4572, along with mutants of *mtrA*, *mtrB*, and *omcB*/*omcA*, was found to be limited in solid Fe oxide (HFOM) reduction relative to the wild type [13]. Interestingly, our LA network also showed that *omcA*, *omcB*, *mtrA *and *mtrB *genes are connected to *cheA-1*, *mgtE-1*, and SO4572.

### The leading LA-scouting gene *cheA-1*

To study the co-expression pattern between seven ARP genes, we took them as genes X and Y to explore gpl3253_cia using the LA system as shown in block 1.2 of Figure 1. The outputs of a short list of 20 genes Z with the best LA scores from the positive and the negative ends are given in Additional file 1. For X = *omcA*, *mtrA*, *omcB*, or *mtrB*, Y = *gspF*, the gene Z with the highest score *LA*(*X*, *Y*|*Z*) is *cheA-1*. In addition, *cheA-1 *also appears in the outputs of (*omcA*, *gspF*), (*mtrA*, *gspF*), (*omcB*, *gspF*), and (*mtrB*, *gspF*) with significant *P *values (Table 1). As one anonymous referee pointed out, genes *cymA*, *mtrA*, *mtrB *and *omcB *were controlled by a global transcriptional regulator CRP [41,42]. We conducted LA analysis using *mtrB*, *omcB*, *mtrA *and *crp *(SO0624) as the lead and found *cheA-1 *again (Additional file 2).

**Table 1:** Liquid association for a positive LA-scouting gene Z (= *cheA-1*).

**X**	**Y**	**Z**	**LA score**	XY Corr (High)*	**XY Corr (Low)^†^**	***P *value**	**Place^‡^**
*mtrA*	*gspF*	*cheA-1*	0.3755	0.4750	-0.2788	0.0001	1
*omcB*	*gspF*	*cheA-1*	0.3765	0.4418	-0.2808	0.0001	1
*mtrB*	*gspF*	*cheA-1*	0.3673	0.5123	-0.1856	0.0002	1
*omcA*	*gspF*	*cheA-1*	0.3185	0.3933	-0.3217	0.0008	1
*omcB*	*gspD*	*cheA-1*	0.2617	0.5016	0.0480	0.0048	6
*mtrB*	*gspD*	*cheA-1*	0.2164	0.4909	0.2799	0.0149	8
*mtrA*	*gspD*	*cheA-1*	0.2034	0.5095	0.1325	0.0163	9
*omcB*	*mtrB*	*cheA-1*	0.2622	0.9718	0.8416	0.0060	15
*omcA*	*gspD*	*cheA-1*	0.1550	0.4553	0.0851	0.0614	16

*The correlation between X and Y in the high *cheA-1 *conditions. ^†^The correlation between X and Y in the low *cheA-1 *conditions. ^‡^The place on the positive end is held by Z.

### *cheA-3*-ARP gene-initiated liquid association search identifies *cheA-1*

In the *S. oneidensis *MR-1, *cheA-3 *gene was necessary for chemotactic behavior [31]. It would be interesting to know how *cheA-3 *may be associated with the electron transport, as shown in block 1.4 of Figure 1, we take *cheA-3*, *omcB*, *mtrA*, *omcA*, *mtrB *and *gspF *as the lead to explore gpl3253_cia. Interestingly, we find the gene *cheA-1 *among the leading 20 positive LA-scouting genes (Table 2). Because *cheA-3 *was essential for chemotactic responses to anaerobic electron acceptors [31], this provided additional evidence about the suggested role of *cheA-1 *as discussed above.

**Table 2:** *cheA-3*-ARP gene-initiated liquid association search identifies *cheA-1*.

**X**	**Y**	**Z**	**LA score**	XY Corr (High)*	**XY Corr (Low)^†^**	***P *value**	**Place^‡^**
*cheA-3*	*omcB*	*cheA-1*	0.2970	0.7545	0.4977	0.0018	3
*cheA-3*	*mtrA*	*cheA-1*	0.2321	0.7804	0.5394	0.0070	3
*cheA-3*	*mtrB*	*cheA-1*	0.2494	0.8241	0.6263	0.0077	5
*cheA-3*	*omcA*	*cheA-1*	0.1732	0.6860	0.4945	0.0474	5
*cheA-3*	*gspF*	*cheA-1*	0.2435	0.9016	0.4166	0.0088	15

*The correlation between X and Y in the high *cheA-1 *conditions. ^†^The correlation between X and Y in the low *cheA-1 *conditions. ^‡^The place on the positive end is held by Z.

### The leading LA-scouting gene *mgtE-1*

Moving to block 1.5 of Figure 1, we find *mgtE-1 *among the leading 20 positive LA-scouting genes when taking *cheA-3*, *omcA*, *mtrA *and *omcB *as the lead (Table 3). Interestingly, as shown in block 1.6, a positive LA-scouting gene *mgtE-1 *at the first place is found after taking *mtrA*, *mtrB*, *omcA *and *omcB *as X and Y (Table 3). As one referee pointed out, genes *mtrB*, *mtrA*, *omcB *and *omcA *are located in a cluster and may be part of the same operon. On the other hand, *gspD*, *gspE *(SO0167) and *gspF *are in another cluster. Interestingly, *mgtE-1 *resulted from the LA analysis on gene pairs in the first cluster, while *cheA-1 *resulted from the LA analysis on gene pairs from different clusters.

**Table 3:** Liquid association for a positive LA-scouting gene Z (= *mgtE-1*).

**X**	**Y**	**Z**	**LA score**	XY Corr (High)*	**XY Corr (Low)^†^**	***P *value**	**Place^‡^**
*omcB*	*mtrA*	*mgtE-1*	0.3216	0.9927	0.9780	0.0003	1
*omcB*	*mtrB*	*mgtE-1*	0.3232	0.9723	0.8796	0.0005	1
*mtrA*	*mtrB*	*mgtE-1*	0.2793	0.9750	0.9404	0.0014	1
*omcA*	*omcB*	*mgtE-1*	0.2511	0.9780	0.9929	0.0042	1
*omcA*	*mtrB*	*mgtE-1*	0.2244	0.9465	0.8814	0.0095	1
*omcA*	*cheA-3*	*mgtE-1*	0.1624	0.7812	0.4578	0.0449	7
*mtrA*	*cheA-3*	*mgtE-1*	0.2087	0.7959	0.5375	0.0105	11
*omcB*	*cheA-3*	*mgtE-1*	0.2480	0.8017	0.5158	0.0043	13

*The correlation between X and Y in the high *mgtE-1 *conditions. ^†^The correlation between X and Y in the low *mgtE-1 *conditions. ^‡^The place on the positive end is held by Z.

### *mgtE-1*-initiated genome-wide liquid association search identifies *coxG*

We treat *mgtE-1 *as the query gene X and evaluate the LA score for every pair of genes (Y, Z) at block 1.7 of Figure 1. We found both genes Y = *coxG *(encoded cytochrome *c *oxidase) and Z = SO0790 (encoded hypothetical protein) at the first place on the positive end. Moving to block 1.8, we also found *cheA-1 *with the largest positive LA score after taking *coxG*, *mtrA*, *mtrB*, *omcA *and *omcB *as X and Y. Within the interior of cell aggregates, aggregate formation may establish the ecological conditions to enhance anaerobic metabolism [43]. Under aerobic-aggregated conditions, *coxG*, haem *c *biosynthesis genes (including SO4520, *ctaB*, *hemB-1 *and *hemN*), anaerobic electron transfer genes (*mtrF *and *mtrD*) and Na-translocating NADH-quinone reductase genes (*nqrA-1 *and *nqrB-1*) were all upregulated [43]. From the analysis performed at block 1.9 of Figure 1, the gene with the largest positive LA score turns out to be SO4572 after taking genes (including *ctaB*, SO4520, *hemN *and *mtrD*) involved in anaerobic respiration as X, Y. Our result provided new insights on the regulation of the genes (including *cheA-1*, *mgtE-1 *and SO4572) that may influence the ability to respire anaerobically in aerobic environments.

### Nitrous oxide (a potent greenhouse gas) study using liquid association

Nitrate reductase (NapA) reduces nitrate (NO_3_^-^) to nitrite (NO_2_^-^). After that, nitrite respiration may proceeds in two different ways [44]. In respiratory denitrification, nitrite is reduced sequentially to nitric oxide (NO), nitrous oxide (N_2_O), and dinitrogen (N_2_) involving nitrous oxide reductase (Nos). Alternatively, nitrite can also be reduced to ammonium (NH_4_^+^) by the nitrite reductase (NrfA) and Cruz-García *et al*. showed that anaerobic cultures of MR-1 grown with nitrate displayed sequential reduction of nitrate to nitrite and then to ammonium [44]. However, the authors also reported the unexpected detection of nitrous oxide and dinitrogen at the same time. The MR-1 genome includes five *nos *genes: *nosA*, *nosL*, *nosD*, *nosF *and *nosY*. From the analysis performed at block 1.10 of Figure 1, we can find *cheA-1 *and SO4572 among the leading 20 positive LA-scouting genes after taking *nosF*, *nosD *and *napA *as X and Y to explore gpl3253_cia (Table 4). The results suggested that little nitrous oxide and dinitrogen detected in Cruz-García *et al*.'s experiment might be produced by the complex regulatory mechanism between *nos *genes, *napA*, SO4572 and *cheA-1*. MR-1 chemotaxis to nitrate and nitrite was reported in the literature [33]. This further supports the scenario of *napA*, *cheA-1 *and SO4572 involvement in affecting N_2_O emission by *S*. *oneidensis *MR-1.

**Table 4:** *nos *gene-*napA*-initiated liquid association search identifies *cheA-1 *and SO4572.

**X**	**Y**	**Z**	**LA score**	XY Corr (High)*	**XY Corr (Low)^†^**	***P *value**	**Place^‡^**
*nosF*	*napA*	*cheA-1*	0.3684	0.7970	-0.4956	0.00002	5
*nosD*	*napA*	*cheA-1*	0.2059	0.8548	0.6178	0.01347	12
*nosF*	*napA*	SO4572	0.3404	0.6923	0.2732	0.00008	17

*The correlation between X and Y in the high Z conditions. ^†^The correlation between X and Y in the low Z conditions. ^‡^The place on the positive end is held by Z.

As suggested by one referee, we used computer to select one thousand pairs of genes randomly from the pool of anaerobic respiration-irrelevant genes (about 4000 genes) and conduct the LA analysis to find out how likely *cheA-1 *and SO4572 will appear as the leading mediator gene by chance. It turns out that *cheA-1 *was detected only 24 times and SO4572 was detected 6 times. Thus statistically, the chance is only 2.4% and 0.6% respectively that our findings might be an artifact.

## Discussion and Conclusions

**Figure 3:**
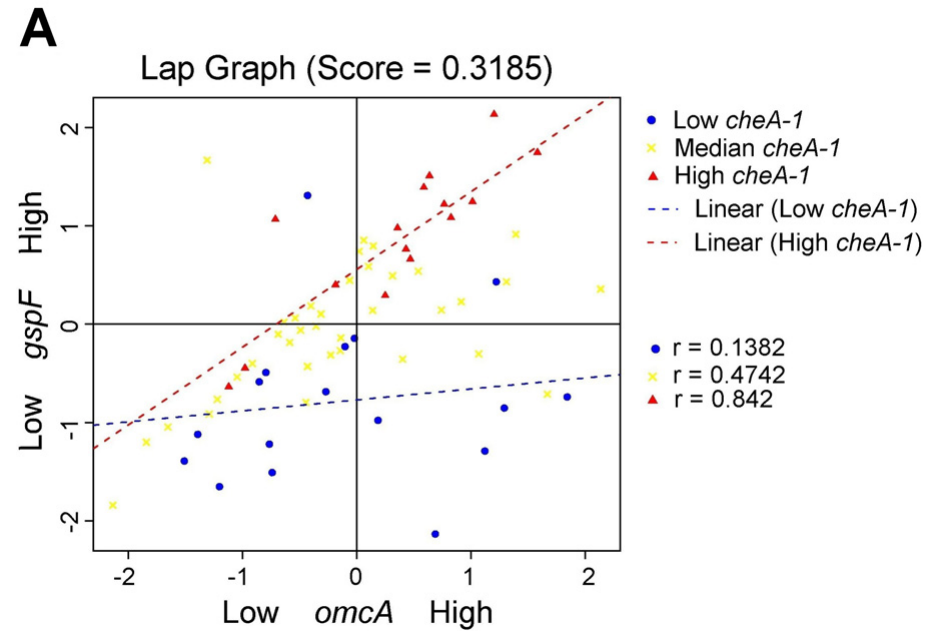
**Liquid association pair (LAP) plot for genes (*omcA,gspF)* as mediated by *cheA-1*.** When *cheA-1* is upregulated (red triangles), a positive association is seen. The correlation disappears as the expression of *cheA-1* is low (blue dots). The LA score is 0.3185.

**Figure 4:**
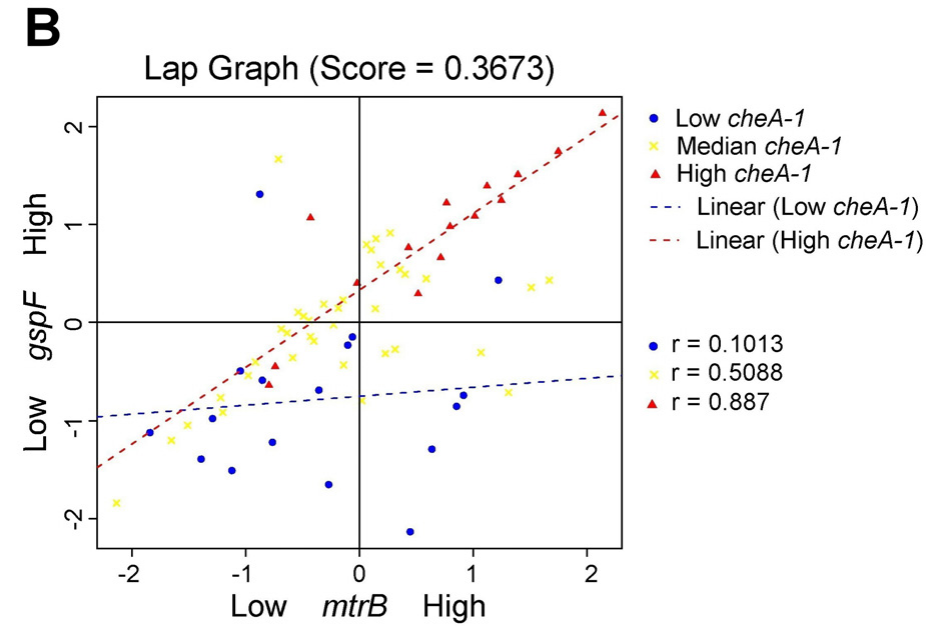
**Liquid association pair (LAP) plot for genes (*mtrB,gspF*) as mediated by *cheA-1*.** When *cheA-1* is upregulated (red triangles), a positive association is seen. The correlation disappears as the expression of cheA-1 is low (blue dots). The LA score is 0.3673.

**Figure 5:**
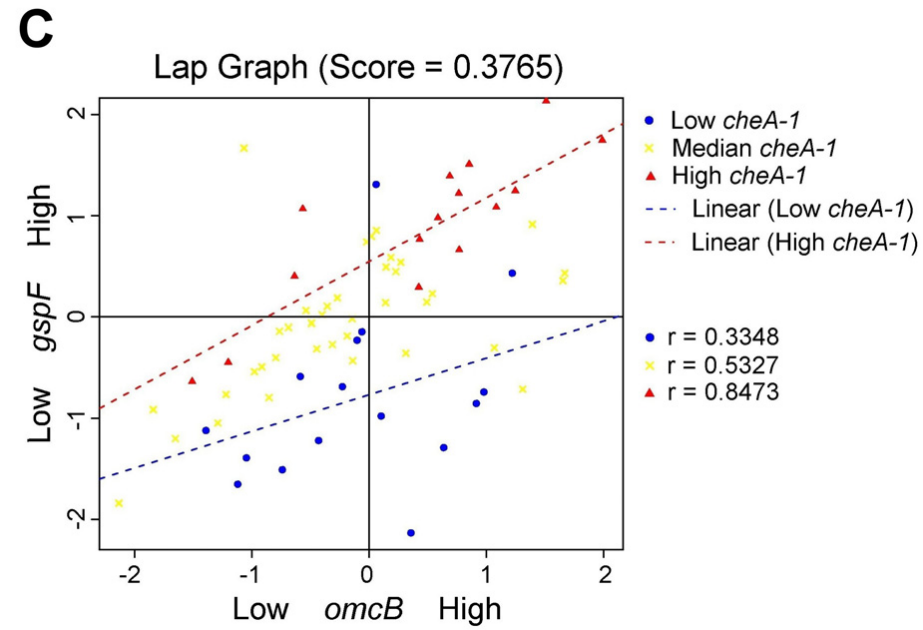
**Liquid association pair (LAP) plot for genes (*omcB,gspF*) as mediated by *cheA-1***. When *cheA-1* is upregulated (red triangles), a positive association is seen. The correlation disappears as the expression of cheA-1 is low (blue dots). The LA score is 0.3765.

**Figure 6:**
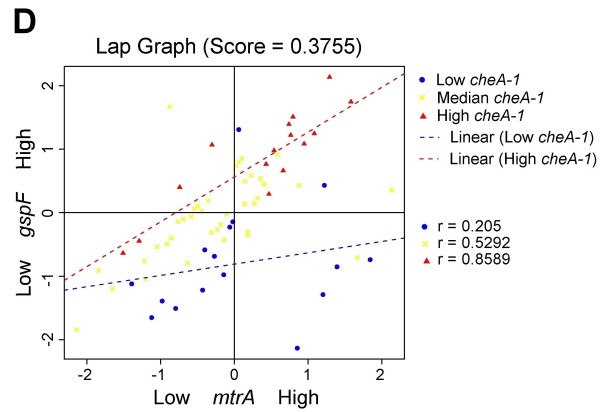
**Liquid association pair (LAP) plot for genes (*mtrA,gspF*) as mediated by *cheA-1*. ** When *cheA-1* is upregulated (red triangles), a positive association is seen. The correlation disappears as the expression of cheA-1 is low (blue dots). The LA score is 0.3755.

All LA plots are easy to create online using our LA system. Figure 3 shows the coexpression pattern change between *omcA *and *gspF *as mediated by gene *cheA-1*. When the expression level of *cheA-1 *is high (conditions represented by red triangles), a strong positive correlation is seen between *omcA *and *gspF *(r = 0.842). When *cheA-1 *is low (blue dots), the association is much decreased (r = 0.1382). A similar interpretation can be given to the LA activity for (*mtrB*, *gspF*), (*mtrA*, *gspF*), and (*omcB*, *gspF*) with *cheA-1 *being the positive scouting gene (Figure 4, 5 and 6).

We examined the experimental conditions associated with the differential coexpression pattern found by LA more closely. In Figure 3, the low expression of *cheA-1 *(blue dots) occurred on the conditions when MR-1 was incubated after a temperature downshift from 30°C to 8°C over a period of 40-80 min and the conditions at 30°C over a period of 60 min after the ionizing radiation (IR) exposure (40 Gy). In contrast, the high expression of *cheA-1 *(red triangles) tended to occur earlier in response to environmental stress: over a period of 5-20 min after the downshift of temperature, and over a period of 20 min after IR exposure. Putting together, our result showed that the up-regulation of the positive LA-scouting gene *cheA-1 *enhanced the co-expression strength of *omcA*, *mtrA*, *omcB*, *mtrB *and *gspF*, thereby increasing the entire electron-flow efficiency. This early response of gene regulation may be an important factor for the survival of MR-1 under environmental stress.

Furthermore, in *S. oneidensis *MR-1, *cheA-3 *gene was essential for chemotactic responses to anaerobic electron acceptors [31]. The LA search initiated by pairing *cheA-3 *with ARP genes identifies *cheA-1 *(Table 2). In addition, Baraquet *et al*. showed that at least one major (SO2240) and four minor (SO3282, SO3642, SO3890 and SO4454) methyl-accepting chemotaxis proteins are involved in energy taxis. We also found *cheA-1 *among the leading 20 positive LA-scouting genes when taking ARP genes and SO2240, SO3282, and SO4454 as the lead (Additional file 3). Our bioinformatic results suggest that *che *and several ARP genes (also including *petC *and SO1415, see Additional file 4) are important for the proper functioning of the mechanisms underlying electron acceptor chemotaxis. Based on the assistance of LA analysis, investigators may design experiments to demonstrate that *cheA-1 *may play a role in optimizing chemotactic behavior. For instance, researchers might study the *cheA-1 *mutant of MR-1 under IR exposure (40 Gy) and/or cold shock (a temperature downshift from 30°C to 8 or 15°C) because our microarray data were extracted from two series GSE3876 (under IR exposure) and GSE4489 (under cold shock).

The slow biotransformation rate of substrates to electrons has been a bottleneck in MFC performance [45]. Applying LA system, we are able to find previously unknown relationship between chemotaxis and electron transfer. Thus our study has the potential of helping researchers to break the internal metabolic limitation of the microbes for the MFC efficiency improvement. It is also noteworthy that there are several statistical methods that may extend the LA system for more complex interaction analysis [26,46-48].

## Methods

We extracted expression profiles of *S. oneidensis *MR-1 from series GSE3876, GSE4489, and GSE7973 in GEO. GSE3876 contained 20 conditions profiled over a period of 1 h after the 40 Gy IR exposure [49]. GSE4489 contained 60 conditions profiled after a temperature downshift from 30°C to 8°C or 15°C over a period of 160 min [50]. GSE7973 consisted of 8 conditions profiled transcriptomic differences between *arcA *(about aerobic respiration control) knockout mutant and wild-type under aerobic or anaerobic environments [51].

### Liquid association analysis

One basic mode of applying LA method is to set X = name of gene A, Y = name of gene B, Z = any gene. The computer will search the database and find a small set of genes Z that are most influential in mediating the correlation pattern between genes A and B. If an increase in Z is associated with an increase in the correlation of (X, Y), then gene Z is a positive LA-scouting gene for (X, Y), and a positive liquid association score *LA*(*X*, *Y*|*Z*) is assigned to quantify the strength of LA. The pair (X, Y) is called a positive LA pair (LAP) of Z. Similarly, a negative LA-scouting gene can be defined if an increase in Z is associated with a decrease in the correlation of (X, Y), and the LA score *LA*(*X*, *Y*|*Z*) is negative. Consequently, when comparing the low with the high expression levels of a negative LA-scouting gene, the scouted LAP is likely to change from being coexpressed to being contraexpressed. For a positive LA-scouting gene, the change goes in the opposite direction: from contraexpression to coexpression [22].

Because the conditions in our dataset come from three different experiments (IR exposure, cold shock, and *arcA *deletion mutant), a normal score transformation on each gene profile for each GEO series was performed individually first. After transformation, we use the formulae, *LA*(*X*, *Y*|*Z*) = (X_1_Y_1_Z_1_+...+X_88_Y_88_Z_88_)/88, to compute the LA score [22,28]. The LAP3 website [52] was developed to enhance the online computation of LA. This website also creates LA graphs, performs standard correlation analysis, reports *P *value, and provides gene ontology (GO) terms [53] of resulting genes of both positive LA-scouting genes (TOP list of Z) and negative LA-scouting genes (BOT list of Z). All annotations in this study were extracted from NCBI or GO database.

## Abbreviations

ARP: anaerobic respiratory plasticity; OM: outer membrane; MFCs: microbial fuel cells; T2S: type II secretion system; CDSs: protein-encoding open reading frames; LA: liquid association; *che*: chemotaxis; IR: ionizing radiation; LAP: LA pair; GO: gene ontology.

## Authors' contributions

SKT, GW, SY, and KCL conducted LA analysis. SKT and KCL wrote the paper. All authors read and approved the final manuscript.

## Supplementary Material

Additional file 1 **Liquid association of 21 LAPs related electron transfer pathways**. This file contains a table listing liquid association of 21 LAPs related electron transfer pathways. Click here for file

Additional file 2 **ARP gene-*crp*-initiated liquid association search identifies *cheA-1 *and *mgtE-1***. This file contains a table showing *cheA-1 *and *mgtE-1 *are among the leading 20 positive LA-scouting genes when taking *mtrB*, *omcB*, *mtrA *and *crp *as the lead. Click here for file

Additional file 3 **ARP gene-chemoreceptor gene-initiated liquid association search identifies *cheA-1***. This file contains a table showing *cheA-1 *is among the leading 20 positive LA-scouting genes when taking *gspF*, *omcB*, *mtrA*, *mtrB*, *omcA*, *gspD*, SO3282, SO4454 and SO2240 as the lead. Click here for file

Additional file 4 **Liquid association search identifies *cheA-1 *and *mgtE-1***. This file contains a table showing *cheA-1 *and *mgtE-1 *would be identified when taking *gspF*, *mtrA*, *omcB*, *gspD*, *omcA*, *petC *and SO1415 as the lead. *petC *(SO0610) and SO1415 are not clustered with the genes (SO1776-9 and SO0166-8) in the genome. Click here for file
