# Supplementary material for: Genome-wide expression links the electron transfer pathway of Shewanella oneidensis to chemotaxis
Source: BMC Genomics. 2010 May 21;11:319. doi: 10.1186/1471-2164-11-319 (PMC2886065; doi:10.1186/1471-2164-11-319)
Supplement: Additional file 1 — Liquid association of 21 LAPs related electron transfer pathways. This file contains a table listing liquid association of 21 LAPs related electron transfer pathways. [file 1471-2164-11-319-S1.DOC]

**Additional file 1**

| Liquid association of 21 LAPs related electron transfer pathways | | | | | |
| --- | --- | --- | --- | --- | --- |
|  | TOP (positive) | |  | BOT (negative) | |
| LAP (X,Y) | Z | LA score |  | Z | LA score |
| *mtrA,gspF* | *cheA-1* | 0.3755 |  | SO0474 | -0.4287 |
|  | SO1729 | 0.3486 |  | SO4044 | -0.4005 |
|  | SO1131 | 0.3419 |  | SO4518 | -0.3605 |
|  | SO3074 | 0.3326 |  | SO2532 | -0.3601 |
|  | SO1266 | 0.3131 |  | SO2000 | -0.3471 |
|  | SO2458 | 0.3100 |  | *bcp* | -0.3427 |
|  | SO0094 | 0.3077 |  | SO2051 | -0.3377 |
|  | SO0336 | 0.3071 |  | SO1455 | -0.3276 |
|  | SO2883 | 0.3063 |  | SO2837 | -0.3223 |
|  | SO2770 | 0.3061 |  | SO0838 | -0.3221 |
|  | SO0757 | 0.3055 |  | SO4546 | -0.3198 |
|  | SO0100 | 0.3008 |  | SO2235 | -0.3178 |
|  | SO2150 | 0.3006 |  | SO3777 | -0.3156 |
|  | SO3481 | 0.3001 |  | SO0149 | -0.3135 |
|  | SO2502 | 0.2992 |  | SO0707 | -0.3114 |
|  | SO2151 | 0.2985 |  | SO4192 | -0.3100 |
|  | SO2977 | 0.2980 |  | SO4588 | -0.3052 |
|  | SO0853 | 0.2956 |  | SO1883 | -0.3028 |
|  | SO4167 | 0.2947 |  | SO0785 | -0.3015 |
|  | SO2868 | 0.2903 |  | *rpmA* | -0.3002 |
| *omcB,gspF* | *cheA-1* | 0.3765 |  | SO0474 | -0.3695 |
|  | SO3074 | 0.3567 |  | SO4044 | -0.3593 |
|  | SO2883 | 0.3458 |  | SO4518 | -0.3571 |
|  | SO1729 | 0.3365 |  | SO2532 | -0.3384 |
|  | SO2770 | 0.3330 |  | SO0838 | -0.3077 |
|  | SO0100 | 0.3292 |  | SO0707 | -0.3063 |
|  | SO1131 | 0.3289 |  | SO2954 | -0.3038 |
|  | SO2458 | 0.3267 |  | SO2000 | -0.3025 |
|  | SO2856 | 0.3154 |  | SO0785 | -0.3021 |
|  | SO0853 | 0.3148 |  | SO3573 | -0.2998 |
|  | SO0793 | 0.3118 |  | SO4352 | -0.2987 |
|  | SO3888 | 0.3118 |  | SO2051 | -0.2977 |
|  | *rseA* | 0.3062 |  | SO0683 | -0.2881 |
|  | SO0726 | 0.3048 |  | SO3777 | -0.2861 |
|  | SO1266 | 0.3039 |  | SO4588 | -0.2803 |
|  | SO0094 | 0.3037 |  | SO1455 | -0.2777 |
|  | SO3481 | 0.3012 |  | SO2602 | -0.2770 |
|  | SO2868 | 0.3006 |  | SO3201 | -0.2748 |
|  | SO3515 | 0.3002 |  | *rpmA* | -0.2741 |
|  | *ifcA-1* | 0.2972 |  | SO1537 | -0.2734 |
| *mtrB,gspF* | *cheA-1* | 0.3673 |  | SO4518 | -0.3998 |
|  | SO3074 | 0.3668 |  | SO4044 | -0.3918 |
|  | SO1131 | 0.3331 |  | SO2532 | -0.3775 |
|  | SO2770 | 0.3322 |  | SO4192 | -0.3529 |
|  | SO4379 | 0.3304 |  | *exbB1* | -0.3362 |
|  | SO4572 | 0.3291 |  | *bcp* | -0.3319 |
|  | SO2502 | 0.3275 |  | SO2235 | -0.3296 |
|  | SO1266 | 0.3258 |  | *hugA* | -0.3291 |
|  | SO0094 | 0.3244 |  | SO0149 | -0.3265 |
|  | SO3481 | 0.3237 |  | SO3408 | -0.3236 |
|  | SO2883 | 0.3215 |  | SO1537 | -0.3202 |
|  | *rseA* | 0.3167 |  | SO1883 | -0.3180 |
|  | SO2458 | 0.3094 |  | SO4352 | -0.3136 |
|  | SO2133 | 0.3090 |  | SO0707 | -0.3128 |
|  | SO4040 | 0.3073 |  | SO2051 | -0.3088 |
|  | SO1057 | 0.3059 |  | SO3777 | -0.3086 |
|  | SOA0095 | 0.3038 |  | SO0683 | -0.3080 |
|  | SO0537 | 0.2986 |  | SO1189 | -0.3079 |
|  | SO3888 | 0.2974 |  | SO3860 | -0.3039 |
|  | *aqpZ* | 0.2963 |  | *rpmA* | -0.3020 |
| *omcA,gspF* | *cheA-1* | 0.3185 |  | SO4044 | -0.3726 |
|  | SO2883 | 0.3115 |  | SO0474 | -0.3552 |
|  | SO2458 | 0.2738 |  | SO2532 | -0.3185 |
|  | SO1131 | 0.2689 |  | SO4518 | -0.3116 |
|  | SO2502 | 0.2674 |  | SO2000 | -0.3056 |
|  | *mvaB* | 0.2661 |  | SO4561 | -0.3008 |
|  | *rseA* | 0.2649 |  | SO1455 | -0.2963 |
|  | SO1266 | 0.2628 |  | SO4192 | -0.2873 |
|  | SO2770 | 0.2621 |  | SO0838 | -0.2870 |
|  | SO1729 | 0.2610 |  | SO4546 | -0.2856 |
|  | SO3074 | 0.2554 |  | *ssb* | -0.2855 |
|  | SO3888 | 0.2545 |  | SO2051 | -0.2834 |
|  | SO2856 | 0.2539 |  | SO0785 | -0.2824 |
|  | SO3481 | 0.2533 |  | SO3402 | -0.2818 |
|  | SO2151 | 0.2527 |  | *bcp* | -0.2773 |
|  | SO2868 | 0.2513 |  | SO2669 | -0.2631 |
|  | SO0853 | 0.2490 |  | SO0961 | -0.2627 |
|  | *hrpA* | 0.2490 |  | SO2144 | -0.2622 |
|  | SO1693 | 0.2487 |  | SO0707 | -0.2618 |
|  | *acs* | 0.2483 |  | SO0149 | -0.2595 |
| *omcA,omcB* | *mgtE-1* | 0.2511 |  | SO2574 | -0.3483 |
|  | SO0705 | 0.2488 |  | SO0475 | -0.3399 |
|  | SO4506 | 0.2371 |  | SO1029 | -0.3107 |
|  | SO4663 | 0.2370 |  | SO2892 | -0.3077 |
|  | SO0851 | 0.2275 |  | SO1721 | -0.2988 |
|  | SO3885 | 0.2228 |  | SO2227 | -0.2945 |
|  | *hypF* | 0.2228 |  | SO0646 | -0.2933 |
|  | SO4052 | 0.2218 |  | SO0517 | -0.2932 |
|  | SO3721 | 0.2209 |  | SO1713 | -0.2925 |
|  | SO3515 | 0.2200 |  | SO1477 | -0.2912 |
|  | SO2666 | 0.2183 |  | SO4609 | -0.2911 |
|  | SO2520 | 0.2120 |  | SO2826 | -0.2911 |
|  | *dcuB* | 0.2107 |  | SO1504 | -0.2862 |
|  | SO2119 | 0.2085 |  | SO2080 | -0.2862 |
|  | SO1238 | 0.2076 |  | SO3370 | -0.2851 |
|  | SO3278 | 0.2075 |  | SO2079 | -0.2843 |
|  | SO3755 | 0.2025 |  | SO2010 | -0.2809 |
|  | SO3001 | 0.2008 |  | SO1572 | -0.2801 |
|  | SO0792 | 0.1996 |  | SO3140 | -0.2783 |
|  | *hoxK* | 0.1994 |  | SO1955 | -0.2777 |
| *omcA,mtrA* | SO4663 | 0.3567 |  | SO4609 | -0.4166 |
|  | SO2692 | 0.3140 |  | *ccmF-2* | -0.3989 |
|  | SO4464 | 0.2922 |  | SO2574 | -0.3975 |
|  | SO3721 | 0.2718 |  | SO0475 | -0.3952 |
|  | SO4431 | 0.2629 |  | SO0517 | -0.3943 |
|  | SO0851 | 0.2626 |  | SO3725 | -0.3801 |
|  | SO0852 | 0.2625 |  | SO1572 | -0.3767 |
|  | SO3278 | 0.2544 |  | *glgA* | -0.3753 |
|  | SO4701 | 0.2438 |  | SO1721 | -0.3697 |
|  | SO4506 | 0.2404 |  | *cysH* | -0.3673 |
|  | SO3001 | 0.2398 |  | SO1029 | -0.3670 |
|  | SO1238 | 0.2392 |  | SO1504 | -0.3650 |
|  | SO0563 | 0.2388 |  | SO4180 | -0.3616 |
|  | SO3716 | 0.2369 |  | SO1477 | -0.3594 |
|  | SO2666 | 0.2363 |  | SOA0003 | -0.3578 |
|  | SO0792 | 0.2355 |  | SO2227 | -0.3511 |
|  | SO0705 | 0.2350 |  | SO1713 | -0.3511 |
|  | SO3885 | 0.2316 |  | SO2713 | -0.3507 |
|  | *hoxK* | 0.2306 |  | SO2077 | -0.3496 |
|  | *mgtE-1* | 0.2288 |  | SO2826 | -0.3495 |
| *omcA,mtrB* | *mgtE-1* | 0.2244 |  | SO2574 | -0.3464 |
|  | SO4663 | 0.2102 |  | *nqrF-2* | -0.3190 |
|  | SO4506 | 0.2089 |  | *sdhA* | -0.3105 |
|  | SO2666 | 0.2017 |  | SO3725 | -0.3103 |
|  | SO3885 | 0.2015 |  | SO0517 | -0.3097 |
|  | SO3515 | 0.1962 |  | SO1537 | -0.3093 |
|  | SO2692 | 0.1946 |  | *glgA* | -0.3088 |
|  | SO3721 | 0.1918 |  | *cysH* | -0.3027 |
|  | SO2862 | 0.1916 |  | SO3140 | -0.2997 |
|  | *hypF* | 0.1912 |  | *nqrD-2* | -0.2911 |
|  | SO4464 | 0.1898 |  | SO1029 | -0.2910 |
|  | SO0705 | 0.1852 |  | SOA0077 | -0.2884 |
|  | *dcuB* | 0.1842 |  | SO1713 | -0.2870 |
|  | SO4691 | 0.1771 |  | *gltA* | -0.2867 |
|  | SO1238 | 0.1760 |  | SO1572 | -0.2792 |
|  | SO4052 | 0.1727 |  | SO3420 | -0.2790 |
|  | SO2822 | 0.1687 |  | SO2227 | -0.2786 |
|  | SO3001 | 0.1666 |  | *sucA* | -0.2780 |
|  | SO2119 | 0.1649 |  | SO1340 | -0.2774 |
|  | SO4051 | 0.1649 |  | SO3724 | -0.2771 |
| *omcA,gspD* | SO3888 | 0.2150 |  | *sdhA* | -0.2682 |
|  | SO3721 | 0.1864 |  | *sdhB* | -0.2413 |
|  | SOA0057 | 0.1797 |  | SO3063 | -0.2395 |
|  | SO0676 | 0.1795 |  | *ptsO* | -0.2153 |
|  | SO4426 | 0.1744 |  | SO4342 | -0.2086 |
|  | *yfiA-1* | 0.1730 |  | SO3975 | -0.2074 |
|  | SO0825 | 0.1717 |  | *nqrF-2* | -0.2038 |
|  | SO3307 | 0.1694 |  | *nqrB-2* | -0.1962 |
|  | SO3283 | 0.1688 |  | *nqrC-2* | -0.1950 |
|  | SO1432 | 0.1630 |  | SOA0051 | -0.1942 |
|  | SO3059 | 0.1608 |  | *glgA* | -0.1935 |
|  | SO2862 | 0.1588 |  | SO2253 | -0.1933 |
|  | SO1996 | 0.1560 |  | SO1210 | -0.1928 |
|  | SO3972 | 0.1552 |  | SO0743 | -0.1921 |
|  | *queA* | 0.1550 |  | SO1321 | -0.1905 |
|  | *cheA-1* | 0.1550 |  | *petB* | -0.1897 |
|  | SO2685 | 0.1538 |  | SO2170 | -0.1895 |
|  | *hoxK* | 0.1510 |  | SO0456 | -0.1895 |
|  | *ifcA-2* | 0.1506 |  | SO3026 | -0.1890 |
|  | *nrfC* | 0.1492 |  | *ahpF* | -0.1890 |
| *omcA,cymA* | SO2053 | 0.3202 |  | SO0724 | -0.3505 |
|  | SO4701 | 0.2913 |  | *pstS* | -0.3492 |
|  | SO0851 | 0.2845 |  | SOA0147 | -0.3409 |
|  | SO3378 | 0.2801 |  | SO0507 | -0.3310 |
|  | SO0180 | 0.2723 |  | SO1622 | -0.3170 |
|  | SO0867 | 0.2710 |  | SO0607 | -0.3122 |
|  | SO0384 | 0.2700 |  | SO4242 | -0.3106 |
|  | SO1768 | 0.2691 |  | SO1836 | -0.3105 |
|  | SO0187 | 0.2574 |  | SO1152 | -0.3073 |
|  | SO0353 | 0.2496 |  | SO2670 | -0.3048 |
|  | SO0387 | 0.2440 |  | *melA* | -0.3008 |
|  | *thiC* | 0.2418 |  | SO4630 | -0.2967 |
|  | SO4151 | 0.2411 |  | SO2685 | -0.2956 |
|  | SO2115 | 0.2373 |  | SO3045 | -0.2949 |
|  | SO1407 | 0.2297 |  | SO1598 | -0.2938 |
|  | SO2553 | 0.2263 |  | SO1697 | -0.2934 |
|  | SO4663 | 0.2189 |  | SO3018 | -0.2933 |
|  | SO0708 | 0.2177 |  | SO2892 | -0.2912 |
|  | SO1728 | 0.2176 |  | SO2568 | -0.2908 |
|  | SO1492 | 0.2153 |  | SO0124 | -0.2869 |
| *omcB,mtrA* | *mgtE-1* | 0.3216 |  | SO0475 | -0.4064 |
|  | SO0705 | 0.3201 |  | SO1029 | -0.3752 |
|  | SO4663 | 0.3179 |  | SO2892 | -0.3745 |
|  | SO3515 | 0.3121 |  | SO2574 | -0.3712 |
|  | SO0851 | 0.3099 |  | SO2080 | -0.3547 |
|  | SO3721 | 0.3052 |  | SO2826 | -0.3539 |
|  | SO4506 | 0.2971 |  | SO1713 | -0.3536 |
|  | SO4052 | 0.2893 |  | SO2227 | -0.3515 |
|  | SO2119 | 0.2835 |  | SO1477 | -0.3512 |
|  | SO2666 | 0.2756 |  | SO2079 | -0.3489 |
|  | SO4431 | 0.2734 |  | SO0517 | -0.3453 |
|  | *hypF* | 0.2711 |  | SO2970 | -0.3411 |
|  | SO3755 | 0.2641 |  | SO4609 | -0.3391 |
|  | SO4464 | 0.2641 |  | SO1537 | -0.3337 |
|  | SO2977 | 0.2625 |  | SO2820 | -0.3335 |
|  | SO3001 | 0.2597 |  | SO3370 | -0.3329 |
|  | SO4691 | 0.2581 |  | SO1721 | -0.3326 |
|  | SO3716 | 0.2576 |  | SO0646 | -0.3307 |
|  | SO0867 | 0.2558 |  | SO4196 | -0.3270 |
|  | *dcuB* | 0.2557 |  | SO1572 | -0.3267 |
| *omcB,mtrB* | *mgtE-1* | 0.3232 |  | SO1537 | -0.3868 |
|  | SO4506 | 0.3154 |  | SO2574 | -0.3718 |
|  | SO4691 | 0.3118 |  | *sdhA* | -0.3565 |
|  | SO3515 | 0.3069 |  | *nqrF-2* | -0.3359 |
|  | *hypF* | 0.2906 |  | *gltA* | -0.3327 |
|  | *dcuB* | 0.2833 |  | *nqrB-2* | -0.3287 |
|  | SO4052 | 0.2791 |  | *nqrD-2* | -0.3264 |
|  | SO2862 | 0.2740 |  | SO2961 | -0.3241 |
|  | SO0154 | 0.2740 |  | SO3140 | -0.3232 |
|  | SO2666 | 0.2740 |  | SO1271 | -0.3224 |
|  | SO2654 | 0.2716 |  | SO1029 | -0.3212 |
|  | SO0705 | 0.2691 |  | *petA* | -0.3193 |
|  | SO0503 | 0.2668 |  | nqrC-2 | -0.3148 |
|  | SO4426 | 0.2651 |  | SO3725 | -0.3127 |
|  | *cheA-1* | 0.2622 |  | SO3370 | -0.3123 |
|  | SO2977 | 0.2604 |  | *petB* | -0.3113 |
|  | SO3885 | 0.2582 |  | SO2266 | -0.3105 |
|  | SO4169 | 0.2565 |  | SO2079 | -0.3079 |
|  | SO3721 | 0.2562 |  | SO3420 | -0.3075 |
|  | SO2656 | 0.2528 |  | *sdhB* | -0.3054 |
| *omcB,gspD* | SO3888 | 0.3027 |  | SO3063 | -0.3046 |
|  | SO3515 | 0.2846 |  | *sdhA* | -0.3012 |
|  | SO4426 | 0.2779 |  | *sdhB* | -0.2690 |
|  | SO0676 | 0.2748 |  | SO3975 | -0.2476 |
|  | SO3721 | 0.2663 |  | SOA0051 | -0.2404 |
|  | *cheA-1* | 0.2617 |  | SO1271 | -0.2386 |
|  | SO1432 | 0.2522 |  | SO2602 | -0.2367 |
|  | SO3755 | 0.2520 |  | SO4342 | -0.2351 |
|  | SO0825 | 0.2456 |  | SO0838 | -0.2320 |
|  | *queA* | 0.2435 |  | SO0456 | -0.2318 |
|  | SO2675 | 0.2410 |  | SO3573 | -0.2295 |
|  | SO0705 | 0.2349 |  | *nqrC-2* | -0.2279 |
|  | SO0216 | 0.2341 |  | *ptsO* | -0.2244 |
|  | SO3329 | 0.2339 |  | *nqrF-2* | -0.2237 |
|  | SO2862 | 0.2336 |  | *thrA* | -0.2228 |
|  | SO2119 | 0.2334 |  | *petB* | -0.2216 |
|  | SOA0057 | 0.2333 |  | SO1210 | -0.2176 |
|  | SO4691 | 0.2317 |  | *rplA* | -0.2174 |
|  | *mgtE-1* | 0.2316 |  | *nqrB-2* | -0.2173 |
|  | SO2770 | 0.2301 |  | SO2717 | -0.2152 |
| *omcB,cymA* | SO0851 | 0.3260 |  | SO0724 | -0.3797 |
|  | SO0867 | 0.3178 |  | SO4630 | -0.3609 |
|  | SO0384 | 0.3134 |  | *pstS* | -0.3347 |
|  | SO1768 | 0.3086 |  | *melA* | -0.3294 |
|  | SO4151 | 0.2990 |  | SO3356 | -0.3203 |
|  | SO2053 | 0.2843 |  | SO1152 | -0.3199 |
|  | SO3378 | 0.2806 |  | SO0124 | -0.3189 |
|  | SO0353 | 0.2783 |  | SO1951 | -0.3186 |
|  | *thiC* | 0.2707 |  | SO2892 | -0.3178 |
|  | SO1728 | 0.2699 |  | SOA0147 | -0.3161 |
|  | SO0387 | 0.2678 |  | SO0924 | -0.3122 |
|  | *leuD* | 0.2672 |  | SO0507 | -0.3064 |
|  | SO0180 | 0.2667 |  | SO2080 | -0.3044 |
|  | SO2553 | 0.2569 |  | SO1622 | -0.3044 |
|  | SO4701 | 0.2560 |  | SO2670 | -0.3022 |
|  | SO2525 | 0.2452 |  | SO0607 | -0.3011 |
|  | SO2656 | 0.2428 |  | SO4242 | -0.3004 |
|  | SO2143 | 0.2416 |  | SO1955 | -0.3003 |
|  | SO1408 | 0.2401 |  | SO1836 | -0.2977 |
|  | SO0708 | 0.2373 |  | *cspD* | -0.2968 |
| *mtrA,mtrB* | *mgtE-1* | 0.2793 |  | SO2574 | -0.3983 |
|  | SO3721 | 0.2702 |  | SO3725 | -0.3618 |
|  | SO4506 | 0.2701 |  | SO1537 | -0.3505 |
|  | SO2666 | 0.2582 |  | SO1029 | -0.3495 |
|  | SO3515 | 0.2576 |  | *nqrD-2* | -0.3435 |
|  | *dcuB* | 0.2533 |  | *nqrF-2* | -0.3402 |
|  | *hypF* | 0.2487 |  | *cysH* | -0.3395 |
|  | SO2862 | 0.2450 |  | SO1713 | -0.3350 |
|  | SO4691 | 0.2384 |  | *sdhA* | -0.3326 |
|  | SO4663 | 0.2375 |  | SO2079 | -0.3319 |
|  | SO3885 | 0.2369 |  | SO0517 | -0.3268 |
|  | SO4431 | 0.2359 |  | SO2297 | -0.3266 |
|  | SO4464 | 0.2344 |  | SO3140 | -0.3258 |
|  | SO3001 | 0.2340 |  | SO2266 | -0.3252 |
|  | SO4169 | 0.2323 |  | *glgA* | -0.3185 |
|  | SO4052 | 0.2294 |  | SO2227 | -0.3160 |
|  | SO0705 | 0.2280 |  | SO2826 | -0.3152 |
|  | SO4426 | 0.2277 |  | *hscB* | -0.3121 |
|  | SO4051 | 0.2261 |  | *cysT-1* | -0.3116 |
|  | SO2656 | 0.2210 |  | *gltA* | -0.3101 |
| *mtrA,gspD* | SO3721 | 0.2429 |  | *sdhA* | -0.2832 |
|  | SO3888 | 0.2426 |  | SO3063 | -0.2752 |
|  | SO4426 | 0.2414 |  | *sdhB* | -0.2592 |
|  | SO3515 | 0.2281 |  | SO4342 | -0.2342 |
|  | SO0676 | 0.2145 |  | SO0743 | -0.2324 |
|  | SO2675 | 0.2086 |  | SO2170 | -0.2321 |
|  | SO3755 | 0.2079 |  | SO0456 | -0.2313 |
|  | SO2119 | 0.2037 |  | *ptsO* | -0.2271 |
|  | *cheA-1* | 0.2034 |  | SO3192 | -0.2233 |
|  | SO3329 | 0.2034 |  | SO1210 | -0.2212 |
|  | *mgtE-1* | 0.2028 |  | SO1271 | -0.2192 |
|  | SO2862 | 0.1962 |  | *petB* | -0.2189 |
|  | *queA* | 0.1923 |  | SO1319 | -0.2178 |
|  | SO1146 | 0.1897 |  | SO2602 | -0.2165 |
|  | SO0705 | 0.1869 |  | SO3975 | -0.2164 |
|  | SO2770 | 0.1843 |  | SOA0051 | -0.2156 |
|  | SO0737 | 0.1836 |  | *gltA* | -0.2134 |
|  | SO3283 | 0.1832 |  | *thrA* | -0.2121 |
|  | *hoxK* | 0.1831 |  | *mreD* | -0.2102 |
|  | SOA0057 | 0.1799 |  | SO4322 | -0.2099 |
| *mtrA,cymA* | SO0851 | 0.3427 |  | *pstS* | -0.3658 |
|  | SO0384 | 0.3250 |  | SOA0147 | -0.3528 |
|  | SO0867 | 0.3198 |  | SO2080 | -0.3506 |
|  | SO2553 | 0.2869 |  | SO1152 | -0.3505 |
|  | SO4663 | 0.2831 |  | SO2568 | -0.3464 |
|  | SO0180 | 0.2808 |  | SO0724 | -0.3426 |
|  | SO4701 | 0.2766 |  | SO0124 | -0.3418 |
|  | SO1768 | 0.2673 |  | SO1622 | -0.3389 |
|  | SO1407 | 0.2646 |  | SO4242 | -0.3361 |
|  | SO3378 | 0.2526 |  | SO2892 | -0.3351 |
|  | SO2053 | 0.2452 |  | SO2670 | -0.3330 |
|  | SO4151 | 0.2442 |  | SO0543 | -0.3323 |
|  | SO2525 | 0.2441 |  | SO1477 | -0.3319 |
|  | SO0353 | 0.2420 |  | SO0475 | -0.3315 |
|  | SO0387 | 0.2418 |  | SO4609 | -0.3300 |
|  | SO3073 | 0.2366 |  | *melA* | -0.3296 |
|  | SO1408 | 0.2340 |  | SO2826 | -0.3289 |
|  | *leuD* | 0.2324 |  | SO2460 | -0.3248 |
|  | SO2119 | 0.2279 |  | SO0652 | -0.3226 |
|  | SO1596 | 0.2275 |  | SO4537 | -0.3177 |
| *mtrB,gspD* | SO3888 | 0.2506 |  | *sdhA* | -0.3440 |
|  | SO0963 | 0.2455 |  | *sdhB* | -0.3022 |
|  | SO2770 | 0.2366 |  | *nqrB-2* | -0.2769 |
|  | SO4426 | 0.2335 |  | *gltA* | -0.2757 |
|  | *yfiA-1* | 0.2313 |  | *nqrC-2* | -0.2701 |
|  | SO2685 | 0.2193 |  | SO1271 | -0.2690 |
|  | SO2502 | 0.2173 |  | SO2532 | -0.2661 |
|  | *cheA-1* | 0.2164 |  | *nqrF-2* | -0.2651 |
|  | SO0940 | 0.2121 |  | SO1537 | -0.2636 |
|  | SO0941 | 0.2113 |  | *petA* | -0.2633 |
|  | SO2702 | 0.2048 |  | SO0354 | -0.2611 |
|  | SO4492 | 0.2044 |  | *mdh* | -0.2546 |
|  | SO1693 | 0.2027 |  | *cysT-1* | -0.2533 |
|  | SO2693 | 0.1995 |  | SO2602 | -0.2516 |
|  | SO3283 | 0.1992 |  | SO2840 | -0.2484 |
|  | SO0389 | 0.1987 |  | SO3408 | -0.2481 |
|  | SO4592 | 0.1986 |  | SO4280 | -0.2446 |
|  | SO0661 | 0.1982 |  | *ahpC* | -0.2440 |
|  | SOA0095 | 0.1976 |  | *exbB1* | -0.2412 |
|  | SO0325 | 0.1967 |  | *nqrD-2* | -0.2357 |
| *mtrB,cymA* | SO0851 | 0.2887 |  | SO1029 | -0.3023 |
|  | SO0384 | 0.2660 |  | *csrA* | -0.2796 |
|  | SO3378 | 0.2546 |  | SO2010 | -0.2780 |
|  | SO2119 | 0.2521 |  | SO2574 | -0.2747 |
|  | SO0867 | 0.2491 |  | SO2080 | -0.2732 |
|  | SO4663 | 0.2406 |  | SO2826 | -0.2729 |
|  | SO4169 | 0.2349 |  | *melA* | -0.2655 |
|  | SO1768 | 0.2316 |  | SO4242 | -0.2633 |
|  | SO2654 | 0.2262 |  | SO0475 | -0.2615 |
|  | SO0387 | 0.2214 |  | SO1477 | -0.2587 |
|  | SO0180 | 0.2188 |  | SO2892 | -0.2562 |
|  | SO0705 | 0.2162 |  | SO3370 | -0.2548 |
|  | SO2520 | 0.2160 |  | SO3140 | -0.2505 |
|  | SO2553 | 0.2125 |  | SO4609 | -0.2476 |
|  | SO4151 | 0.2125 |  | SO1572 | -0.2474 |
|  | *mexE* | 0.2122 |  | SO0924 | -0.2462 |
|  | SO4431 | 0.2110 |  | SO1412 | -0.2448 |
|  | *mgtE-1* | 0.2103 |  | SO1152 | -0.2369 |
|  | SO1407 | 0.2102 |  | SO4196 | -0.2353 |
|  | SO3073 | 0.2097 |  | SO1598 | -0.2335 |
| *gspF,gspD* | SO2770 | 0.2718 |  | SO2532 | -0.2511 |
|  | SO0367 | 0.2526 |  | SO0707 | -0.2459 |
|  | SO2677 | 0.2440 |  | SO4353 | -0.2349 |
|  | SO2661 | 0.2280 |  | SO0782 | -0.2298 |
|  | SO4572 | 0.2254 |  | SO3573 | -0.2275 |
|  | SO3515 | 0.2236 |  | SO4444 | -0.2251 |
|  | SO4736 | 0.2225 |  | SO1128 | -0.2204 |
|  | SO2660 | 0.2219 |  | SO3571 | -0.2200 |
|  | SO2997 | 0.2210 |  | SO3777 | -0.2148 |
|  | SO3773 | 0.2146 |  | SO2726 | -0.2125 |
|  | *trpG* | 0.2102 |  | SOA0056 | -0.2098 |
|  | SO1890 | 0.2062 |  | SO2602 | -0.2093 |
|  | SO3003 | 0.2055 |  | SO3572 | -0.2090 |
|  | SO2205 | 0.2037 |  | *rpmA* | -0.2039 |
|  | SO0666 | 0.2013 |  | SO2589 | -0.2035 |
|  | SO2695 | 0.1998 |  | SO4743 | -0.2015 |
|  | SO4194 | 0.1987 |  | SO0182 | -0.2000 |
|  | SO2655 | 0.1915 |  | SO3707 | -0.1999 |
|  | SO2983 | 0.1907 |  | SO2550 | -0.1996 |
|  | *ifcA-1* | 0.1907 |  | SO2235 | -0.1994 |
| *gspF,cymA* | SO4650 | 0.2691 |  | SO3326 | -0.2659 |
|  | SO2143 | 0.2308 |  | SO3626 | -0.2473 |
|  | *cysA-2* | 0.2251 |  | SO4630 | -0.2287 |
|  | SO1768 | 0.2247 |  | SO2106 | -0.2257 |
|  | SO1728 | 0.2177 |  | SO2943 | -0.2252 |
|  | *sbp* | 0.2126 |  | SO1951 | -0.2224 |
|  | SO0851 | 0.2122 |  | SO0474 | -0.2181 |
|  | SO2150 | 0.2119 |  | *cydB* | -0.2090 |
|  | *cysA-1* | 0.2088 |  | SO0924 | -0.2080 |
|  | SO1729 | 0.2045 |  | SO0133 | -0.2078 |
|  | SO2862 | 0.2018 |  | SO3816 | -0.2058 |
|  | SO4151 | 0.1951 |  | SO1988 | -0.2036 |
|  | SO0674 | 0.1938 |  | SO3699 | -0.2027 |
|  | *cysN* | 0.1917 |  | SO0574 | -0.2020 |
|  | SO0690 | 0.1912 |  | *ushA* | -0.1996 |
|  | SO0867 | 0.1903 |  | *melA* | -0.1978 |
|  | SO3722 | 0.1865 |  | SO2201 | -0.1937 |
|  | SO0503 | 0.1855 |  | SO2669 | -0.1936 |
|  | *mexE* | 0.1837 |  | *hisC* | -0.1888 |
|  | SO4339 | 0.1835 |  | SO0581 | -0.1886 |
| *gspD,cymA* | SO1073 | 0.2353 |  | SO3326 | -0.1977 |
|  | SO0676 | 0.2343 |  | SO0108 | -0.1861 |
|  | SO3765 | 0.2247 |  | SO2943 | -0.1791 |
|  | SO3888 | 0.2117 |  | SO1951 | -0.1713 |
|  | SO3721 | 0.2092 |  | SO0051 | -0.1685 |
|  | SO3755 | 0.2073 |  | SO1323 | -0.1631 |
|  | SO3475 | 0.1985 |  | *secY* | -0.1621 |
|  | *hoxK* | 0.1945 |  | SO3626 | -0.1616 |
|  | SO2075 | 0.1926 |  | *modC* | -0.1615 |
|  | SO4015 | 0.1876 |  | SO3549 | -0.1614 |
|  | SO3504 | 0.1841 |  | SO3616 | -0.1575 |
|  | SO2119 | 0.1825 |  | SO1993 | -0.1548 |
|  | SO3515 | 0.1822 |  | *melA* | -0.1545 |
|  | SO4058 | 0.1817 |  | SO2280 | -0.1543 |
|  | SO2862 | 0.1806 |  | SO4007 | -0.1501 |
|  | SOA0057 | 0.1802 |  | SO1599 | -0.1440 |
|  | *kdpE* | 0.1788 |  | SO1622 | -0.1432 |
|  | SO1768 | 0.1787 |  | SO1654 | -0.1423 |
|  | SO0772 | 0.1784 |  | *aroQ* | -0.1419 |
|  | SO0851 | 0.1778 |  | SO4242 | -0.1401 |
