# Supplementary material for: Genome-wide expression links the electron transfer pathway of Shewanella oneidensis to chemotaxis
Source: BMC Genomics. 2010 May 21;11:319. doi: 10.1186/1471-2164-11-319 (PMC2886065; doi:10.1186/1471-2164-11-319)
Supplement: Additional file 2 — ARP gene-crp-initiated liquid association search identifies cheA-1 and mgtE-1 . This file contains a table showing cheA-1 and mgtE-1 are among the leading 20 positive LA-scouting genes when taking mtrB, omcB, mtrA and crp as the lead. [file 1471-2164-11-319-S2.DOC]

**Additional file 2**

| ARP gene-*crp*-initiated liquid association search identifies *cheA-1* and *mgtE-1* | | | | | | |
| --- | --- | --- | --- | --- | --- | --- |
| X | Y | Z | LA score | XY Corr^*^ | *P* value | Place^†^ |
| *mtrB* | *crp* | *cheA-1* | 0.3361 | 0.8382 | 0.00010 | 1 |
| *omcB* | *crp* | *cheA-1* | 0.3416 | 0.8561 | 0.00008 | 2 |
| *mtrA* | *crp* | *cheA-1* | 0.2688 | 0.8098 | 0.00073 | 4 |
| *mtrA* | *crp* | *mgtE-1* | 0.2286 | 0.8098 | 0.00234 | 14 |
| *omcB* | *crp* | *mgtE-1* | 0.2679 | 0.8561 | 0.00105 | 19 |

^*^The correlation between X and Y. ^†^The place on the positive end is held by Z.
