# Supplementary material for: Genome-wide expression links the electron transfer pathway of Shewanella oneidensis to chemotaxis
Source: BMC Genomics. 2010 May 21;11:319. doi: 10.1186/1471-2164-11-319 (PMC2886065; doi:10.1186/1471-2164-11-319)
Supplement: Additional file 3 — ARP gene-chemoreceptor gene-initiated liquid association search identifies cheA-1 . This file contains a table showing cheA-1 is among the leading 20 positive LA-scouting genes when taking gspF, omcB, mtrA, mtrB, omcA, gspD, SO3282, SO4454 and SO2240 as the lead. [file 1471-2164-11-319-S3.DOC]

**Additional file 3**

| ARP gene-chemoreceptor gene-initiated liquid association search identifies *cheA-1* | | | | | | |
| --- | --- | --- | --- | --- | --- | --- |
| X | Y | Z | LA score | XY Corr^*^ | *P* value | Place^†^ |
| *gspF* | SO3282 | *cheA-1* | 0.4054 | 0.3930 | 0.00002 | 1 |
| *omcB* | SO4454 | *cheA-1* | 0.3638 | 0.4615 | 0.00054 | 1 |
| *mtrA* | SO4454 | *cheA-1* | 0.3365 | 0.4384 | 0.00059 | 1 |
| *mtrB* | SO4454 | *cheA-1* | 0.3222 | 0.5191 | 0.00205 | 2 |
| *omcA* | SO4454 | *cheA-1* | 0.2760 | 0.3530 | 0.00720 | 2 |
| *gspF* | SO2240 | *cheA-1* | 0.2934 | 0.5350 | 0.00111 | 3 |
| *gspD* | SO3282 | *cheA-1* | 0.1945 | 0.5186 | 0.02063 | 5 |

^*^The correlation between X and Y. ^†^The place on the positive end is held by Z.
