# Supplementary material for: Genome-wide expression links the electron transfer pathway of Shewanella oneidensis to chemotaxis
Source: BMC Genomics. 2010 May 21;11:319. doi: 10.1186/1471-2164-11-319 (PMC2886065; doi:10.1186/1471-2164-11-319)
Supplement: Additional file 4 — Liquid association search identifies cheA-1 and mgtE-1 . This file contains a table showing cheA-1 and mgtE-1 would be identified when taking gspF, mtrA, omcB, gspD, omcA, petC and SO1415 as the lead. petC (SO0610) and SO1415 are not clustered with the genes (SO1776-9 and SO0166-8) in the genome. [file 1471-2164-11-319-S4.DOC]

**Additional file 4**

| Liquid association search identifies *cheA-1* and *mgtE-1* | | | | | |  |
| --- | --- | --- | --- | --- | --- | --- |
| X | Y | Z | LA score | XY Corr^*^ | *P* value | Place^†^ |
| *gspF* | SO1415 | *cheA-1* | 0.3906 | 0.2624 | 0.00005 | 2 |
| *mtrA* | *petC* | *mgtE-1* | 0.3451 | 0.4385 | 0.00005 | 14 |
| *omcB* | *petC* | *mgtE-1* | 0.3738 | 0.4126 | 0.00004 | 15 |
| *gspD* | SO1415 | *cheA-1* | 0.1568 | 0.3946 | 0.04227 | 15 |
| *omcA* | *petC* | *mgtE-1* | 0.2860 | 0.3792 | 0.00163 | 18 |

^*^The correlation between X and Y. ^†^The place on the positive end is held by Z.
